# miR-153/KCNQ4 axis contributes to noise-induced hearing loss in a mouse model

**DOI:** 10.1186/s12576-021-00814-0

**Published:** 2021-09-03

**Authors:** Qin Wang, Wei Li, Cuiyun Cai, Peng Hu, Ruosha Lai

**Affiliations:** Department of Otolaryngology and Head & Neck Surgery, The Second Xiangya Hospital, Central South University, Changsha, 410011 Hunan China

**Keywords:** Sensorineural hearing loss, miR-153, KCNQ4, Noise exposure, OHC loss

## Abstract

Damage to the cochlear sensory epithelium is a key contributor to noise-induced sensorineural hearing loss (SNHL). KCNQ4 plays an important role in the cochlear potassium circulation and outer hair cells survival. As miR-153 can target and regulate KCNQ4, we sought to study the role of miR-153 in SNHL. 12-week-old male CBA/J mice were exposed to 2–20 kHz broadband noise at 96 dB SPL to induce temporary threshold shifts and 101 dB SPL to induce permanent threshold shifts. Hearing loss was determined by auditory brainstem responses (ABR). Relative expression of miR-153 and KCNQ4 in mice cochlea were determined by Real-Time quantitative PCR. miR-153 mimics were co-transfected with wild type or mutated KCNQ4 into HEK293 cells. Luciferase reporter assay was used to validate the binding between miR-153 and KCNQ4. AAV-sp-153 was constructed and administrated intra-peritoneally 24- and 2-h prior and immediately after noise exposure to knockdown miR-153. The KCNQ4 is mainly expressed in outer hair cells (OHCs). We showed that the expression of KCNQ4 in mice cochlea was reduced and miR-153 expression was significantly increased after noise exposure compared to control. miR-153 bound to 3′UTR of KNCQ4, and the knockdown of miR-153 with the AAV-sp-153 administration restored KCNQ4 mRNA and protein expression. In addition, the knockdown of miR-153 reduced ABR threshold shifts at 8, 16, and 32 kHz after permanent threshold shifts (PTS) noise exposure. Correspondingly, OHC losses were attenuated with inhibition of miR-153. This study demonstrates that miR-153 inhibition significantly restores KNCQ4 in cochlea after noise exposure, which attenuates SNHL. Our study provides a new potential therapeutic target in the prevention and treatment of SNHL.

**Supplementary Information:**

The online version contains supplementary material available at 10.1186/s12576-021-00814-0.

## Introduction

Base on a World Health Organization (WHO) 2021 report, approximately 5% of population worldwide need rehabilitation to resolve disabling hearing loss [1]. Hearing loss is mainly divided into three different types: sensorineural hearing loss (SNHL), conductive hearing loss, and mixed hearing loss. SNHL can be caused by inner ear damage or auditory nerve disease, resulting in degeneration of cochlear hair cells, supporting cells and auditory nerve endings. Damage to the cochlear sensory epithelium is a key contributor to SNHL [2]. Noise exposure has been considered as a social health problem in various workplace, such as army, farming, and construction, negatively influencing life quality.

Damage to cochlear hair cells plays an important role in the development of SNHL. Compared with birds and primitive vertebrates, the regenerative capacity of mammalian hair cells is limited, which increases the risk of irreversible hearing loss [3–5]. There are two types of sensory receptor cells in the cochlea of the inner ear that are crucial for hearing: the inner hair cells (IHCs) and the outer hair cells (OHCs). The IHCs are transducers of sound vibrations, whereas the OHCs amplify the sound signal in the cochlea [6]. Long-term exposure to high levels of noise is associated with IHCs and OHCs damage, leading to temporary threshold shifts (TTS) or permanent threshold shifts (PTS).

KCNQ4 (KV7.4), a member of the KCNQ family (Kv7) of voltage-gated potassium channels, is predominantly found in the inner ear and central auditory pathways [7–10]. In the inner ear, KCNQ4 is abundantly expressed in the basolateral membrane of the OHCs, mediating the M-like potassium current I_K,n_ [11–13]. The activity of the KCNQ4 channel plays an important role in maintaining the membrane potential of inner ear hair cells and the K^+^ recycling in the cochlea [14, 15]. KCNQ4 is the main channel of the OHCs to maintain K^+^ homeostasis in the cochlea [9, 10]. The expression of KCNQ4 can also be detected in the vestibular sensory epithelium and the nucleus neurons of certain auditory pathways [9, 16]. It has been reported that human KCNQ4 mutation can cause DFNA2 non-syndromic deafness [7]. Variants in KCNQ4, e.g., c.1044_1051del8 [17], Pro291Leu [18] have also been identified that are involved in autosomal recessive and semi-dominant hearing loss, respectively. However, the physiological mechanisms of KCNQ4 in regulating auditory pathways remain unknown. A study has shown that the 3′-UTR region of KCNQ4 has the binding site of miR-153, which could regulate the expression of KCNQ4 [19]. KCNQ4 has been recognized as one of the most susceptible genes for noise-induced hearing loss [20]. These studies revealed that targeting KCNQ4 via miR-153 could act as a pharmacological approach of treatment and management for SNHL.

Therefore, we hypothesize that miR-153 can target KCNQ4, thereby regulating the survival and improving function of OHCs. The present study is designed to investigate the pattern of miR-153 expression in inner ear hair cells and whether miR-153 and KCNQ4 can independently regulate the survival and function of cochlear hair cells.

## Methods

### Animals

**Fig. 1:**
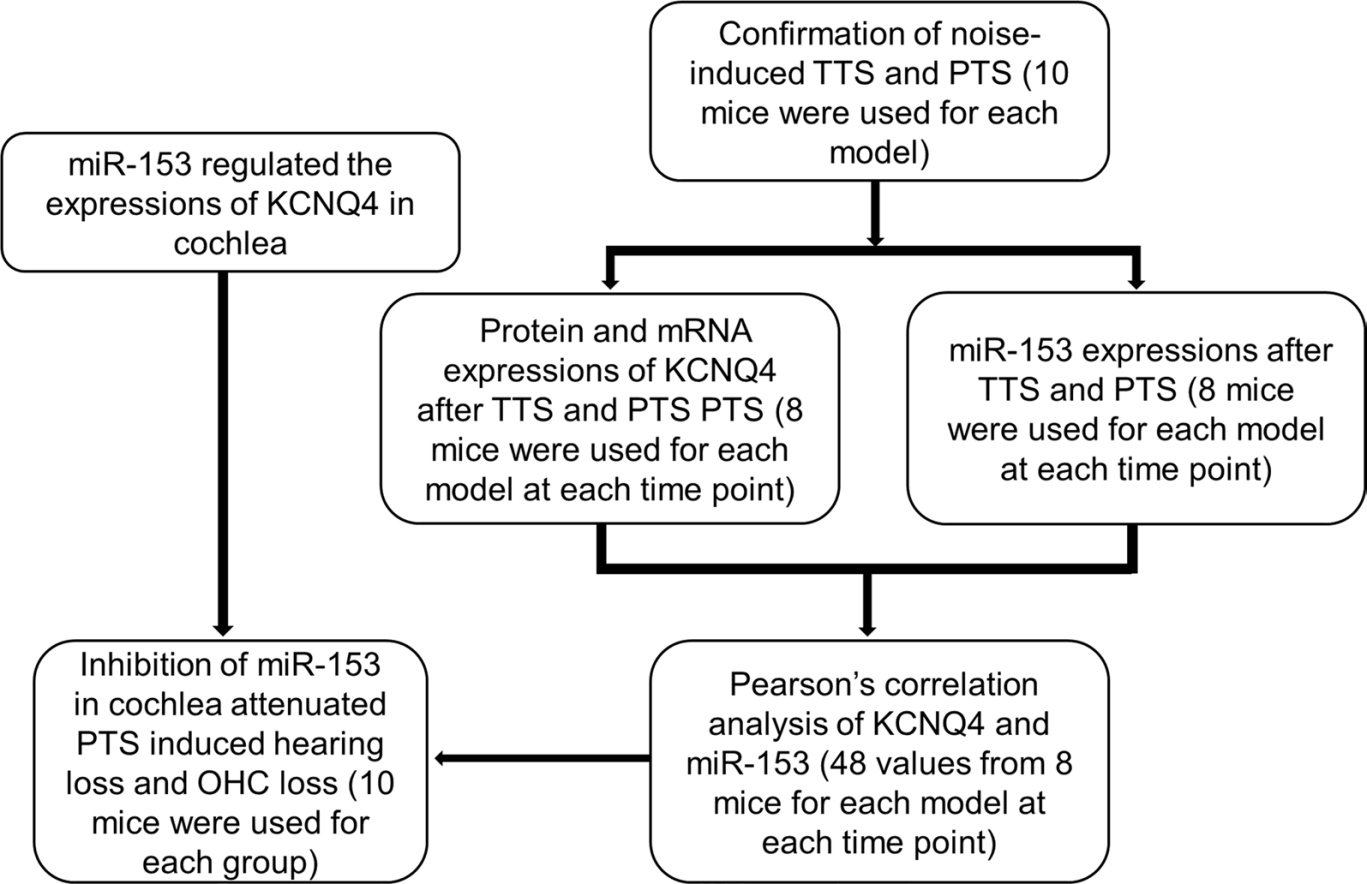
Schematic design of this study

Male CBA/J mice at the age of 12 weeks were housed at 22 °C ± 1 °C under a standard condition 12:12 h light–dark cycle to acclimate for 1 week before the experiments. All animal experiments were approved by the Ethic Committee of the Second Xiangya Hospital, Central South University. The study of the design is presented in Fig. 1.

### Noise exposure

It was reported that exposure of mice to 2–20 kHz broadband noise (BBN) at 92, 94, or 96 dB SPL for 2 h could induces TTS, while intensities of 98–106 dB SPL resulted in PTS and hair cell death without altering the structure of supporting cells and spiral ganglion cells [21]. Therefore, 12-week-old male CBA/J mice were exposed to BBN with a frequency spectrum from 2 to 20 kHz for 2 h at 96-dB SPL to induce TTS, and 101-dB SPL to induce PTS.

### Auditory brainstem responses

Auditory brainstem responses (ABRs) were measured as described in previous published work [22]. 10 ms tone bursts with a 1 ms rise-fall time were presented at 8, 16 and 32 kHz at a rate of 21.1/s. The average response to 1024 stimuli was gained through reducing the intensity in 5 dB intervals near the threshold. Thresholds were estimated based on the lowest stimulus level, where a response was observed and the highest level, where no response was observed. All ABR measurements were conducted by the same person. The ABR measurements were assigned by an expert who was blinded to the treatment conditions.

### Surface preparations and hair cell counts

The cochleae were decalcified with ethylenediaminetetraacetic acid, followed by permeabilizing with 0.3% Triton X in phosphate-buffered saline (PBS) for 15 min at room temperature. Subsequently, cochleae were stained with 100 μL of phalloidin (Life Technology, Carlsbad, CA, USA) containing FITC at 37 °C in the dark for 120 min, followed by incubated with 10 mg/ml 4′,6-diamidino-2-phenylindole (Sigma, St. Louis, MO, USA) for 10 min and mounted on glass slides in 50% glycerol. Cochlear was observed and imaged using an Olympus BX63 microscope (Olympus, Tokyo, Japan). Hair cells including OHCs and IHCs were counted from the apex to the base along the entire length of rat cochlear epithelium. Two positions of the hair cells were calculated, at 10–20 and 65–70% of the whole cochlear duct distance from the apex, approximately aligning with the 7–8 or 32–36 kHz frequency domain. Hair cells were calculated in six mice from each group and were considered to be degenerated if the cell nuclei were absent.

### Vector construction and virus

For construction of miR-153 knockout virus vector, sponge-miR-153 sequence (Additional file 1: Table S1) was cloned into the lentivirus background vector. Adeno-associated virus (AAV)-sponge-153 construct and the vector contained a GFP sequence were driven by the CMV promoter. The lentivirus was concentrated by ultracentrifugation at 3000*g* for 30 min at 4 °C, followed by precipitating with virus precipitation solution (ExCell Bio) for 24 h and resuspended in 30 μL of PBS. The adeno-associated virus AAV-9 contained AAV-sp-153, which were obtained from Hanbio Company (Shanghai, China).

### Drug administration via intra-peritoneal route

0.6 μg AAV-sp-153 was resuspended in 30 μL PBS and administered intra-peritoneally 24- and 2 h prior and immediately after noise exposure. The animals in the experiment designed to observe the evolution of the ABR threshold after noise exposure received the fourth IP injection at 24 h after the noise exposure. The animals were euthanized at 1 h after noise exposure and temporal bones were removed to dissect the cochleae for Real-Time qPCR and western blot analysis.

### Western blot analysis

The mice (*n* = 8 per group) cochleae were collected, and homogenates were pooled for Western blot analysis. The protein concentrations were determine using BCA assay. 10 μg protein were loaded and separated using tris–glycine gel. Proteins were then transferred to polyvinylidene fluoride membrane. Followingly, blots were blocked in 5% non-fat milk for 1 h at room temperature. The blots were incubated with primary antibodies were used against KCNQ4 (1:1000, ab84820, Abcam) and β-actin (1:3000, ab6276, Abcam) in the cold room overnight. The density of target bands was measured using Image J analysis software (JAVA image processing program, NIH, Bethesda, MD), and normalized to β-actin. Each experiment was repeated independently 4 times.

### Real-time qPCR analysis

The mice (*n* = 8 per group) cochleae were collected and homogenized individually for qPCR analysis. The gene expression of *Kcnq4* and miR-153 in cochlea were determined using Real-Time qPCR (RT-qPCR). The following primers were used, as shown in Additional file 1: Table S1. RT-qPCR was performed and calculated using the comparative threshold cycle (Ct) method. Pearson correlation was analyzed using SPSS software.

### Dual luciferase assay

A fragment of the KCNQ4 mRNA 3′UTR (NM_0047000.3) for miR-153 was synthesized and cloned into pLightSwitch_3′UTR luciferase expression reporter vector. The pLightSwitch_3′UTR luciferase expression reporter vector containing no insert was used as negative control for transfection to allow assay normalization. HEK293 cells were co-transfected with luciferase reporters containing wild type or mutant *Kcnq4* 3′UTR with miR-153 mimics or negative control using TransIT-X2. The sequences were shown in Additional file 1: Table S1. After incubation for 24 h, luciferase activities were measured using the LightSwitch Assay Reagent kit (LS010, Active Motif) designed for use with all GoClone reporter plasmids.

### Statistical analysis

The significant difference was carried out by one-way or two-way ANOVA with an indicated post hoc test in GraphPad prism 7. Error bar represents mean ± SD. *P* value smaller than 0.05 was considered statistically significant.

## Results

### Noise exposures induced temporary and permanent hearing loss in adult CBA/J mice

**Fig. 2:**
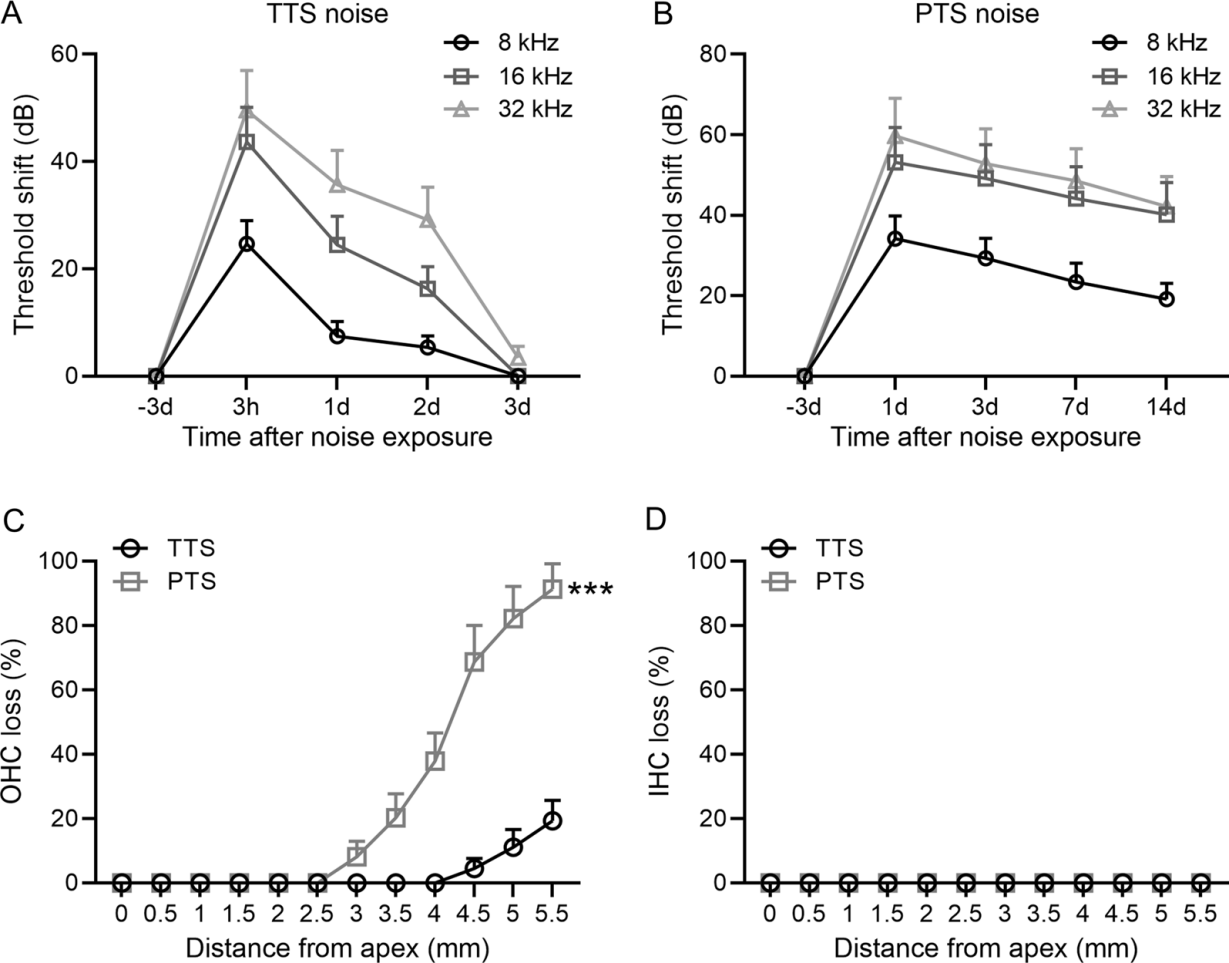
Noise-induced TTS and PTS in adult CBA/J mice. **A** TTS: 96 dB SPL induced significant threshold shifts at 8, 16, and 32 kHz 3 h after exposure with complete recovery by day 3. *n* = 10 at each time. **B** PTS: 101 dB SPL induced significant threshold shifts at 8, 16, and 32 kHz 1 days after noise exposure that are maintained through 14 days. *n* = 10 at each time. **C** Quantification of OHC losses 7 days after noise exposure revealed more OHC loss with PTS compared to TTS. *n* = 10 for each group. **D** Quantification of IHC losses 7 days after noise exposure revealed no IHC loss in both groups. *n* = 10 for each group. Data were presented as mean ± SD. ***P < 0.001 compared to TTS. Two-way ANOVA followed Tukey's multiple comparisons test

Here, we evaluated the noise exposure at 96 dB SPL to induce TTS and at 101 dB SPL to induce PTS. We found that auditory thresholds shifted significantly at 8, 16, and 32 kHz at 3 h after exposure to BBN at 96 dB SPL (Fig. 2A). The auditory thresholds at all three frequencies fully recovered after 3-day exposure, indicating that 96 dB SPL induced TTS only (Fig. 2A). Exposures at 101 dB SPL induced significant threshold shifts at 8, 16, and 32 kHz at 1-day post-exposure, which maintained throughout 14 days (Fig. 2B). After 7-day noise exposure, the loss of hair cell was counted and quantified for TTS and PTS. Our results showed that significantly more OHC losses were observed after 101 dB PTS noise compared to 96 dB TTS noise after 7-day noise exposure (*P* < 0.001, Fig. 2C). However, no IHC loss were found in both TTS and PTS groups (Fig. 2D). Therefore, the 96 dB SPL noise designed as “TTS noise” and the 101 dB SPL noise designed as “PTS noise”.

### KCNQ4 protein levels were reduced after TTS and PTS

**Fig. 3:**
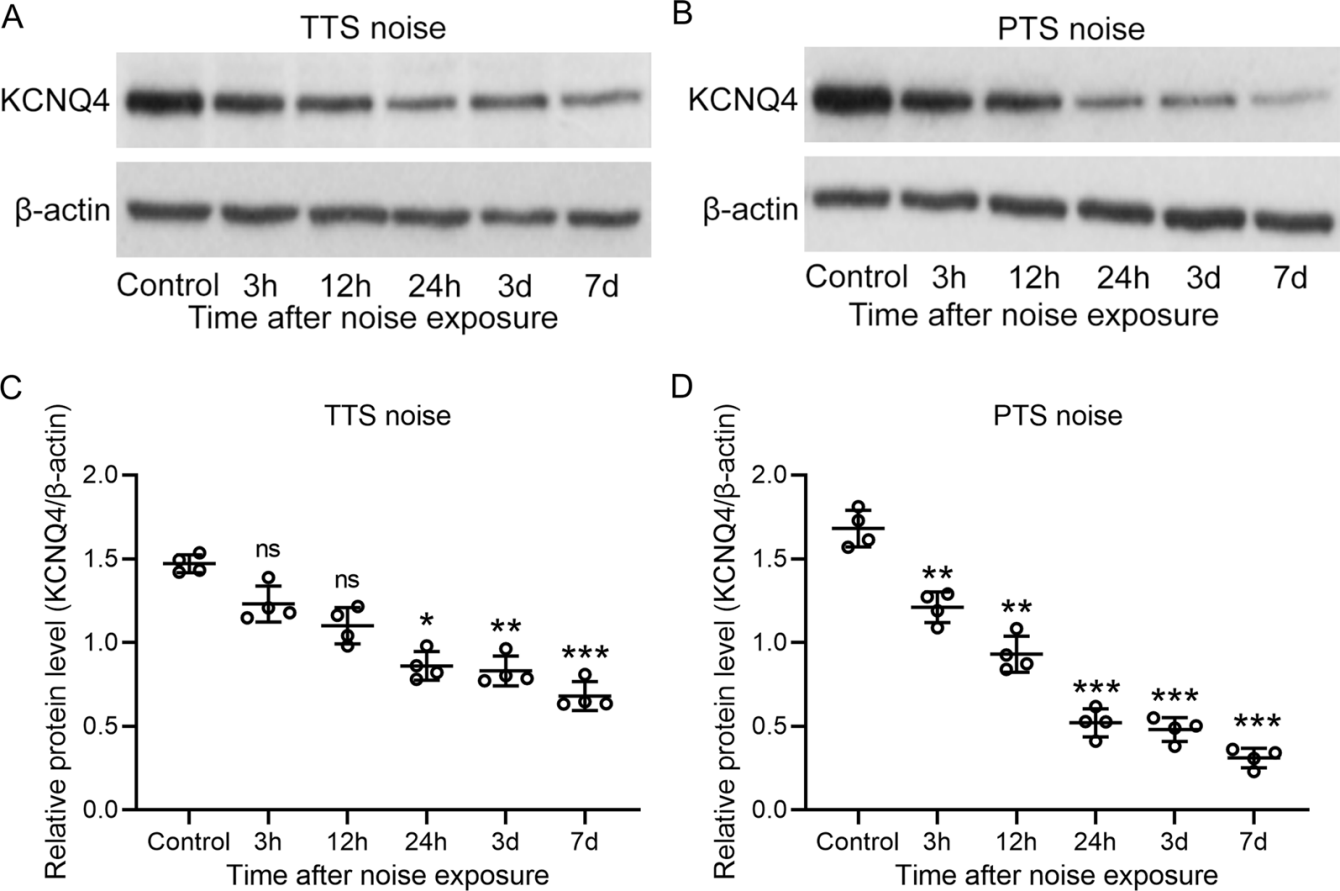
Protein levels of KCNQ4 was reduced after both TTS and PTS. The protein levels of KCNQ4 in cochlea at different time after TTS (**A**) and PTS (**B**) measured by western blotting and normalized to β-actin (**C**, **D**). Data were presented as mean ± SD with all data points showing. *n* = 4 from 8 mice in each group. *P < 0.05, **P < 0.01, ***P < 0.001 and ns means no significance compared to control. One-way ANOVA followed Dunn's multiple comparisons test

KCNQ4, a voltage-gated potassium channel, localized to the OHC of the inner ear. We determined the protein levels of KCNQ4 after TTS and PTS noise exposure by Western blot. We found that the protein levels of KCNQ4 were notably decreased by 41% relative to control after 24 h TTS noise exposure (*P* < 0.05, Fig. 3A, C). Moreover, a greater decrease of protein levels of KCNQ4 were observed after 3-day (44%) and 7-day (54%) TTS noise exposure compared to control (*P* < 0.01, *P* < 0.001, respectively, Fig. 3A, C). The protein levels of KCNQ4 were decreased by 28% relative to control after 3 h PTS noise exposure (*P* < 0.01, Fig. 3B, D). The protein levels of KCNQ4 were greatly reduced after 7-day (82%) PTS noise exposure relative to control (*P* < 0.001, Fig. 3B, D). Taken together, our results suggested that the protein levels of KCNQ4 were significantly reduced in both TTS and PTS groups. KCNQ4 protein levels after PTS noise exposure were markedly lower compared to TTS noise exposure.

### miR-153 was increased and KCNQ4 mRNA expression was reduced after both TTS and PTS

**Fig. 4:**
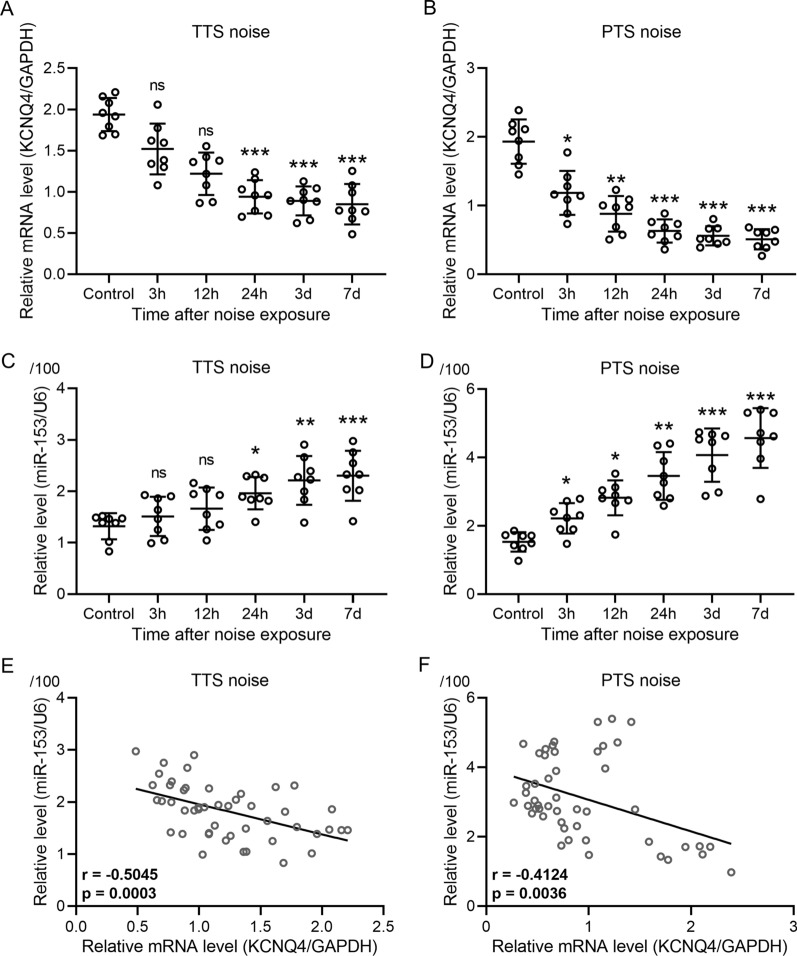
Expression of miR-153 was increased and mRNA expression of *Kcnq4* was reduced after both TTS and PTS. **A**–**D** qRT-PCR was used to measure the expressions of miR-153 and mRNA expression of *Kcnq4* in cochlea at different time after TTS and PTS. Data were presented as mean ± SD with all data points showing. *n* = 8 in each group. *P < 0.05, **P < 0.01, ***P < 0.001 and ns [42] means no significance compared to control. One-way ANOVA followed Dunn's multiple comparisons test. **E**, **F** Pearson’s correlation analysis was carried out to measure the correlations between miR-153 expressions and *Kcnq4* mRNA expressions in cochlea after TTS and PTS

miR-153 is a highly conserved microRNA in mice and human, which has been showed to promote neurogenesis [23]. To determine the role of miR-153 in mice with TTS and PTS noise exposure, we evaluated miR-153 levels and mRNA expression of *KCNQ4* using RT-qPCR. Our results showed that mRNA levels of *KCNQ4* were significantly suppressed in mice after 24 h TTS noise exposure relative to control (*P* < 0.001, respectively, Fig. 4A). In parallel, mRNA levels of *KCNQ4* were markedly reduced after 3 h PTS noise exposure relative to control (*P* < 0.05), and a greater decrease was observed after 24 h PTS noise exposure (*P* < 0.001) and maintained through 7 days (Fig. 4B). In contrast, we found that miR-153 was significantly increased in both TTS (1.7-fold) and PTS (3.0-fold) through 7-day noise exposure compared to control (*P* < 0.001, *P* < 0.001, respectively, Fig. 4C, D). To determine the correlations between expressions of miR-153 and *KCNQ4* mRNA in mice with TTS and PTS noise exposure, we carried out Pearson’s correlation analysis. These findings suggest that miR-153 expression was increased, while *KCNQ4* was reduced after TTS and PTS noise exposure (Fig. 4E, F). The alteration of miR-153 and KCNQ4 were more obvious in PTS compared to TTS.

### miR-153 interacted with KCNQ4 3′-UTR

**Fig. 5:**
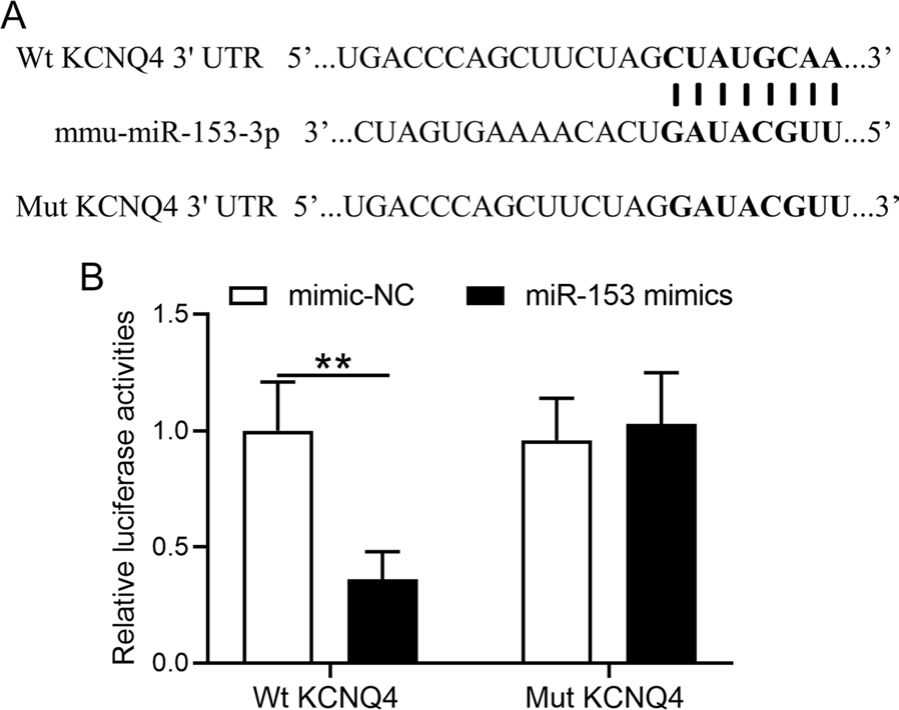
miR-153 targets KCNQ4. **A** Sequences of miRNA and the potential miRNA binding sites at the 3′UTR of KCNQ4. **B** HEK-293T cells were co-transfected with luciferase reporters containing WT and/or mutant KCNQ4 3′-UTR with miR-153 mimics and negative control. After 48 h of incubation, relative luciferase activities were measured. Data were presented as mean ± SD. *n* = 4 for each group. **P < 0.01. Two-way ANOVA followed Sidak's multiple comparisons test

To further determine whether miR-153 regulates KCNQ4 directly, a luciferase reporter was constructed containing a fragment of *KCNQ4* mRNA 3′UTR carrying suspected binding of mmu-miR-153-3p with the wild-type (Wt) 3′UTR region of *KCNQ4* mRNA (Fig. 5A); or a mutated (Mut) 3′-URT of *KCNQ4*. HEK293 cells were co-transfected with luciferase reporters containing wild type or mutant KCNQ4 3′UTR with miR-153 mimics. Our results showed that after 48 h luciferase activity in HEK293 cells of WT KCNQ4 3′-UTR with miR-153 mimics was significantly reduced by 64% when compared to negative control (*P* < 0.01, Fig. 5B). In contrast, luciferase activity in HEK293 cells of mutant KCNQ4 3′-UTR with miR-153 mimics returned to the level of negative control (Fig. 5B), indicating that miR-153 targeted KCNQ4.

### KCNQ4 expression was increased via inhibition of miR-153 in cochlea

**Fig. 6:**
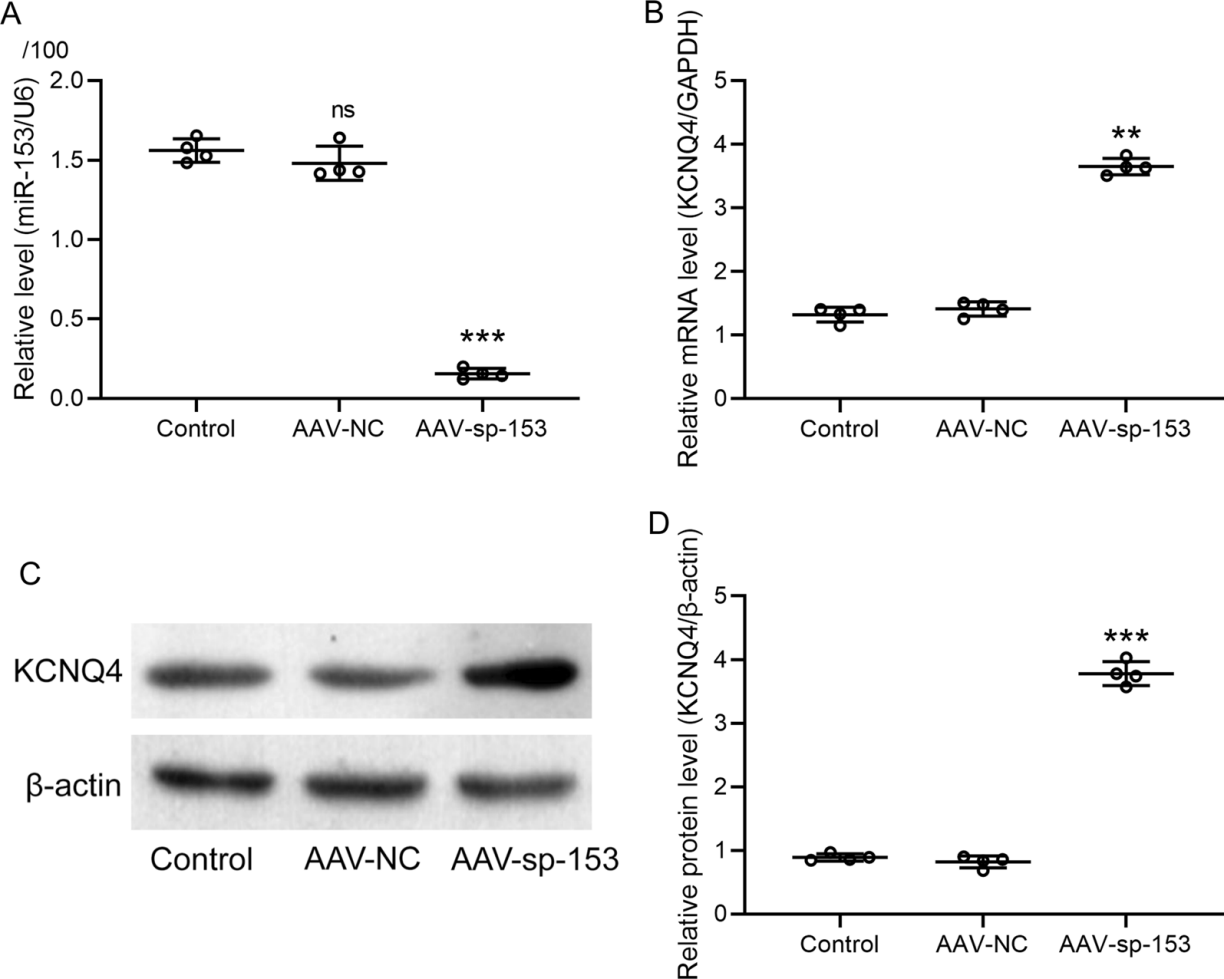
Inhibition of miR-153 increased the expressions of KCNQ4 in cochlea. The expressions of miR-153 (**A**) and mRNA expression of *Kcnq4* in cochlea (**B**) after two administrations of AAV-sp-153 measured by RT-qPCR. The protein levels of KCNQ4 in cochlea after two administrations of AAV-sp-153 (**C**) were measured by western blot and normalized to β-actin (**D**). Data were presented as mean ± SD with all data points showing. *n* = 4 from 8 mice in each group. **P < 0.01, ***P < 0.001 and ns means no significance compared to control. One-way ANOVA followed Dunn's multiple comparisons test

To evaluate the role of miR-153 in regulating KCNQ4 in mice with PTS noise exposure, mice received two administrations of AAV-sp-153 intra-peritoneally to inhibit miR-153 expression. To confirm the transfection efficiency, we measured miR-153 expression in cochlea using RT-qPCR. miR-153 expression was significantly reduced by 90% relative to control (*P* < 0.001, Fig. 6A). Furthermore, we found mRNA level of *KCNQ4* was significantly increased by 2.8-fold after the treatment of AAV-sp-153 compared to control (*P* < 0.01, Fig. 6B). Corresponding with mRNA results, protein level of KCNQ4 was notably increased with the treatment of AAV-sp-153 by 4.2-fold relative to control (*P* < 0.001, Fig. 6C, D). These findings suggest that the treatment of AAV-sp-153 could significantly inhibit miR-153 and upregulate KCNQ4 in cochlea.

### Inhibition of miR-153 attenuated PTS-induced hearing loss and OHC loss

**Fig. 7:**
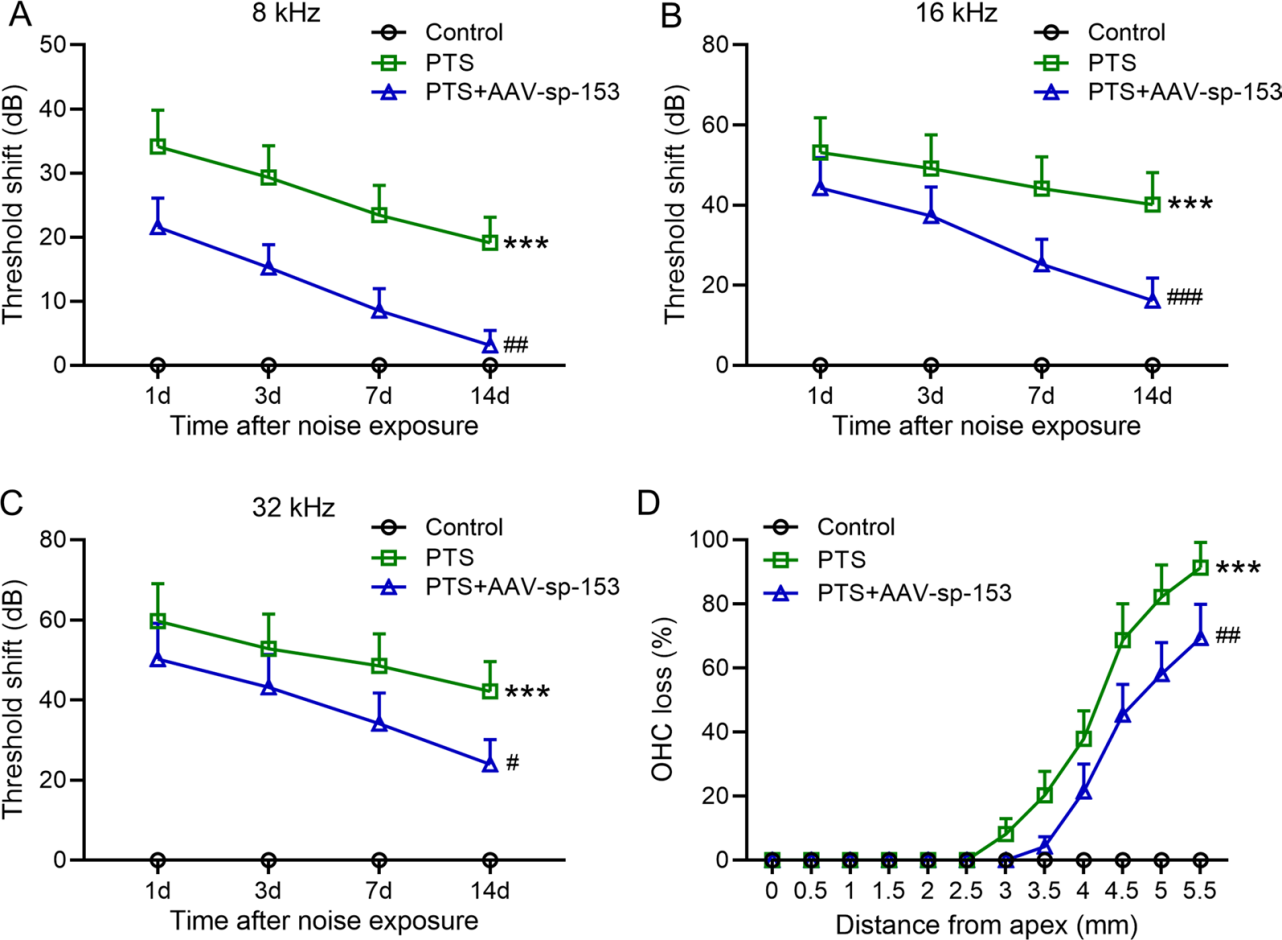
Inhibition of miR-153 in cochlea attenuated PTS induced hearing loss and OHC loss. Auditory brainstem responses were tested at different time after PTS exposure (**A**–**C**) and quantification of OHC losses 7 days after PTS exposure (**D**). *n* = 10 for each group. Data were presented as mean ± SD. ***P < 0.001 compared to control and ^#^*P* < 0.05, ^*##*^*P* < 0.01, ^###^*P* < 0.001 compared to PTS group. Two-way ANOVA followed Tukey's multiple comparisons test

To further investigate the role of miR-153 in PTS induced hearing loss and OHC loss, ABR measurements were performed at 8, 16, 32 kHz after PTS noise exposure. We found inhibition of miR-153 by administration with AAV-sp-153 significantly reduced the thresholds at 8, 16 and 32 kHz throughout 14 days of PTS noise exposure when compared to mice without AAV-sp-153 treatment after PTS exposure (*P* < 0.01, *P* < 0.001, *P* < 0.05, respectively, Fig. 7A–C). In addition, OHC losses began from 2.5 mm from apex after PTS exposure in mice without AAV-sp-153 treatment, whereas 3 mm from apex in mice treated with AAV-sp-153. The amount of OHC losses in mice treated with AAV-sp-153 significantly reduced by 24% compared to mice without treatment (*P* < 0.01, Fig. 7D). These observations indicate that inhibition of miR-153 could attenuate the hearing loss and OHC loss induced by PTS.

## Discussion

Noise-induced SNHL is becoming a major social health problem affecting people’s life quality worldwide. In this study, we demonstrated that KCNQ4 is critical involved in SNHL after TTS and PTS noise exposure. The miR-153 could target KCNQ4 and reduce KCNQ4 expression after TTS and PTS noise exposure. Inhibition of miR-153 showed to attenuate hair cells damage and OHC loss via upregulating KCNQ4 in mice with PTS noise exposure.

Previous studies have demonstrated that exposure of CBA/J mice for 2 h 2–20 kHz BBN at 92, 94, or 96 dB SPL induce TTS, and 98, 100, 102, 104, and 106 dB SPL lead to PTS without altering the structure of supporting cell and spiral ganglion cell [22]. Consistent with previous study [22], we have shown that 96 dB SPL markedly induced auditory threshold shifts at 8, 16 and 32 kHz after 3 h exposure, which was completely recovered 3 days after exposure, indicating that the noise causes TTS only. When exposure to 101 dB SPL, a larger threshold shifts were observed at 8, 16, and 32 kHz at 1 day after exposure. No recovery was observed at all three frequencies after 2-week exposure, suggesting that 101 dB SPL induced PTS in mice. In parallel with these findings, OHC losses with PTS were significantly higher than TTS, whereas no IHC losses was found in both PTS and TTS. OHCs are more susceptible to noise compared to IHCs [24], suggesting that OHCs play a pivotal role in noise-induced SNHL.

KCNQ4 is mainly found in OHCs in the inner ear. Mutations in KCNQ4 have been found in patients with deafness nonsyndromic autosomal dominant 2 (DFNA2) hearing loss [25, 26], which is attributed to progressive SNHL [10, 27, 28]. Studies showed that restoration of KCNQ4 using KCNQ channel openers, retigabine, can reduce OHC degeneration and hearing loss in DFNA2 [29]. However, KCNQ channel openers, such as retigabine, have low efficacy on native channel [29] along with side effects, including urinary retention [30] and skin discoloration [31]. Corresponding with the in vitro study, KCNQ4 knockout mice shows a notable elevation of auditory steady-state response and ABR amplitudes compared to wild-type mice, estimating a more serve hearing loss in KCNQ4 knockout mice [32]. The functional loss of KCNQ4 caused by pharmacological inhibition or gene knockout can lead to OHCs degeneration and progressive SNHL without disrupting vestibular phenotype [27]. In addition, exposure to noise disrupts ion-trafficking system cause the decrease of Na^+^, K^+^-ATPase activity in the cochlear, which may involve the oxidative stress [33]. The elevated reactive oxygen species after noise exposure further cause damage to cochlear tissue, leading to the reduction of KCNQ4 expression [33–35]. Further research found that the absence of KCNQ4 disrupts the potassium ion current of OHCs, subsequently causing depolarization of OHCs and inhibiting OHCs sound amplification [27]. In agreement with these findings, our results detected a significantly decrease of KCNQ4 in mice after TTS or PTS noise exposure. However, contrary to OHCs, IHCs are mostly intact with only slightly depolarization in KCNQ4-deficient mice [27]. These findings highlight the essential role of KCNQ4 in hearing, which could serve as a target in developing the pharmacological treatment for SNHL.

miRNAs are RNA molecules with a length of about 21 to 23 nucleotides that are widely present in eukaryotes, playing an important role in regulating gene expression, cell cycle, and organism development timing [36, 37]. miRNA can effectively target hundreds of genes to modulate the multiple biological pathways in the patient's body that are involved in the pathophysiological process [38]. miRNA binds to the recognition site of the three prime untranslated region (3′UTR) of mRNA, thereby triggering mRNA degradation and inhibiting mRNA transcription and protein translation [39, 40]. Studies have found that down-regulation of miR-183, miR-96 and miR-182 can cause the reduction of hair cells, the loss of semicircular canals and abnormal nerve mounds [41, 42]. Computational in silico analysis revealed that miR-153 putatively binds to the 3′UTR of KCNQ4, which was confirmed by luciferase reporter assay [23]. Study showed that miR-153 was increased in arteries from spontaneously hypertensive rats, while KCNQ4 level was decreased [23]. These findings suggest that miR-153 could serve as a therapeutic target directly binding to 3′UTR of KCNQ4 and attenuating vascular dysfunction in hypertension. In our study, RT-qPCR confirmed that miR-153 expression levels were significantly increased in cochlea after TTS or PTS noise exposure. In contrast, the mRNA levels of *Kcnq4* were notably reduced in both TTS and PTS groups. The Pearson’s correlation analysis indicated that miR-153 was negatively correlated to *Kcnq4*, indicating that miR-153 may potentially regulate KCNQ4 post-transcription. To validate the binding between miR-153 and KCNQ4, a dual luciferase assay was performed to confirm that miR-153 can directly target 3′UTR region of KCNQ4. In this study, we administrated mice with constructed adeno-associated virus AAV-sp-153 to knockdown miR-153. miR-153 expression in cochlea was significantly reduced compared to control, confirming the knockdown of miR-153. In consistent with this result, we observed that the mRNA and protein levels of KCNQ4 were significantly increased in cochlea with the treatment with AAV-sp-153. Our results lead to the conclusion that inhibition of miR-153 increases the mRNA and protein levels of KCNQ4. Regarding to the regulatory role of miR-153 in mice with SNHL, we found that ABR threshold shifts significantly reduced after PTS noise exposure in mice treated with AAV-sp-153 compared to PTS group, implying that inhibition of miR-153 strikingly reduced hearing loss in noise induced SNHL. In addition, we showed that inhibition of miR-153 effectively attenuated OHCs loss after noise exposure. Increasing evidences show that miRNA plays a critical role in regulating gene expression in sensory cells in inner ear, which contributes to the development and progressive of hearing loss [43]. With respect to develop therapeutic approach using miRNA, to identify the miRNA caused hearing loss and downstream target genes is crucially important for developing the treatment for hearing loss [44]. Thus, the safety and efficacy of targeting cells require more research in the future.

## Conclusions

The present study demonstrated that miR-153 can directly bind to 3′UTR region of KCNQ4, thereby causing striking suppression of KCNQ4 in mice after PTS noise exposure. The inhibition of miR-153 effectively restored KCNQ4 in cochlea after noise exposure in mice. Our findings provide a novel approach for the prevention and treatment of noise induced SNHL.

### Supplementary Information

**Additional file 1: Table S1.** Sequence used in this study.

## Data Availability

Data could be obtained upon request to the corresponding author.

## Abbreviations

SNHL: Sensorineural hearing loss
BBN: Broadband noise
OHC: Outer hair cells
IHC: Inner hair cells
ABR: Auditory brainstem responses
PTS: Permanent threshold shifts
