# Supplementary material for: miR-153/KCNQ4 axis contributes to noise-induced hearing loss in a mouse model
Source: J Physiol Sci. 2021 Sep 3;71:28. doi: 10.1186/s12576-021-00814-0 (PMC10718010; doi:10.1186/s12576-021-00814-0)
Supplement: Supplementary file 1 — Additional file 1: Table S1. Sequence used in this study. [file 12576_2021_814_MOESM1_ESM.docx]

**Supplementary materials**

Table S1. Sequence used in this study.

|  | Forward | Reverse |
| --- | --- | --- |
| sponge-miR-153 | AATTCGATCACTTTTACTCTATGCAACCGGATCACTTTTACTCTATGCAAGCGGATCACTTTTACTCTATGCAAG | GATCCTTGCATAGAGTAAAAGTGATCCGCTTGCATAGAGTAAAAGTGATCCGGTTGCATAGAGTAAAAGTGATCG |
| KCNQ4 | GGAAACCCTTCTGTGTCATCGA | TGTGCCCGCAGCTATCACT |
| GAPDH | AGGTCGGTGTGAACGGATTTG | TGTAGACCATGTAGTTGAGGTCA |
| miR-153 | ACACTCCAGCTGGGTTGCATAGTCACAAA | CAGTGCGTGTCGTGGAGT |
| U6 | TCGCACAGACTTGTGGGAGAA | CGCACATTAAGCCTCTATAGTTACTAGG |
| Dual luciferase assay | | |
| Wt KCNQ4 3’UTR | 5’ UGACCCAGCUUCUAGCUAUGCAA 3’ | |
| mmu-miR-153p | 3’ CUAGUGAAAACACUGAUACGUU 5’ | |
| Mut KCNQ4 3’UTR | 5’ UGACCCAGCUUCUAGGAUAGUU 3’ | |
